# Rho-Associated Kinase Inhibitor (Y-27632) Attenuates Doxorubicin-Induced Apoptosis of Human Cardiac Stem Cells

**DOI:** 10.1371/journal.pone.0144513

**Published:** 2015-12-08

**Authors:** Lijuan Kan, Aubrie Smith, Miao Chen, Benjamin T. Ledford, Huimin Fan, Zhongmin Liu, Jia-Qiang He

**Affiliations:** 1 Department of Biomedical Sciences and Pathobiology, College of Veterinary Medicine, Virginia Polytechnic Institute and State University, Blacksburg, Virginia, United States of America; 2 Research Institute of Heart Failure, Shanghai East Hospital of Tongji University, Shanghai, PR China; Northwestern University, UNITED STATES

## Abstract

**Background:**

Recent clinical trials using c-kit^+^ human cardiac stem cells (CSCs) demonstrated promising results in increasing cardiac function and improving quality of life. However, CSC efficiency is low, likely due to limited cell survival and engraftment after transplantation. The Rho-associated protein kinase (ROCK) inhibitor, Y-27632, significantly increased cell survival rate, adhesion, and migration in numerous types of cells, including stem cells, suggesting a common feature of the ROCK-mediated apoptotic pathway that may also exist in human CSCs. In this study, we examine the hypothesis that pretreatment of human CSCs with Y-27632 protects cells from Doxorubicin (Dox) induced apoptosis.

**Methods and Results:**

c-kit^+^ CSCs were cultured in CSC medium for 3–5 days followed by 48hr treatment with 0 to 10μM Y-27632 alone, 0 to 1.0μM Dox alone, or Y-27632 followed by Dox (48hrs). Cell viability, toxicity, proliferation, morphology, migration, Caspase-3 activity, expression levels of apoptotic-related key proteins and c-kit^+^ were examined. Results showed that 48hr treatment with Y-27632 alone did not result in great changes in c-kit^+^ expression, proliferation, Caspase-3 activity, or apoptosis; however cell viability was significantly increased and cell migration was promoted. These effects likely involve the ROCK/Actin pathways. In contrast, 48hr treatment with Dox alone dramatically increased Caspase-3 activity, resulting in cell death. Although Y-27632 alone did not affect the expression levels of apoptotic-related key factors (p-Akt, Akt, Bcl-2, Bcl-xl, Bax, cleaved Caspase-3, and Caspase-3) under basal conditions, it significantly inhibited the Dox-induced increase in cleaved Caspase-3 and reduced cell death under Dox treatment.

**Conclusions:**

We conclude that preconditioning human CSCs with Y-27632 significantly reduces Dox-induced cell death and possibly involves the cleaved Caspase-3 and ROCK/Actin pathways. The beneficial effects of Y-27632 may be applied to stem cell-based therapy to increase cell survival rates after transplantation or to act as a cardiac protective agent for Dox-treated cancer patients.

## Introduction

Cardiovascular disease is the leading cause of morbidity and mortality worldwide. In the USA, nearly 85.6 million adults are affected with at least one type of cardiovascular disease, among which myocardial infarction (MI) causes the highest mortality[1]. Despite advances in medical- and catheter-based therapies for MI, the 1 and 5 year mortality rates for this disease remain as high as 13% and 50%, respectively[1]. Thus, alternative strategies, such as stem cell therapy, are urgently needed [2]. Numerous animal and human studies have demonstrated that stem cells hold great potential to regenerate dead myocardial tissue and induce neovascularization in infarcted areas, thereby, alleviating the underlying cause of heart failure[3].

Among all types of stem cells (*i*.*e*., embryonic stem cells (ESCs), induced pluripotent stem cells (iPSCs), fetal stem cells, and adult stem cells), cardiac stem cells (CSCs) have been found to include several subtypes of stem cells based on their cell surface markers[4]. Examples include cardiosphere-derived cells, Isl1 positive stem cells, Sca-1 positive cells, and c-kit positive stem cells (*i*.*e*., c-kit^+^ CSCs). c-kit^+^ CSCs seem to be one of the most promising cells types used in clinical trials to repair ischemic heart failure, likely because of their cardiac origination and their capability of being auto-transplanted without immunorejection[5]. In animal studies, human c-kit^+^ CSCs demonstrated a considerable ability to differentiate into three cardiac lineages (*i*.*e*., cardiomyocytes, smooth muscle, and endothelial cells) *in vivo* after transplantation into immunosuppressed rats[6] or mice [7], with the transplanted c-kit^+^ CSCs restoring cardiac structure and function[8]. Recently, two clinical trials using autologous human CSCs showed promising results by increasing cardiac function, reducing the amount of scar tissue, and improving the quality of patients’ lives, without any observed safety issues[9, 10]. Unfortunately, most of the animal studies and human clinical trials showed only small or marginal improvements in cardiac function based on echocardiograph and MRI analyses. A detailed analysis of animal models suggested that the major reasons for this marginal efficacy is likely related to low cell survival (due to significant cell death after transplantation), low cell retention, and low cell engraftment and integration into host cardiac tissues following transplantation[11]. Thus, to date, developing an effective approach to prevent cell death after transplantation is one of the most urgent and challenging tasks in the field.

Over the past decade, various methods have been explored to improve cell survival rates, including the application of a pro-survival cocktail, preconditioning the stem cells with growth factors/small chemical compounds/hypoxia culture (*e*.*g*., IGF2, hypoxia culture, and Y-27632), and genetic overexpression of anti-apoptotic genes (*e*.*g*., Bcl-2, HO-1, βadrenergic receptor kinase or pim-1), applications of immunosuppression drug, anti-inflammation, and/or in combination with bioengineered matrices[12]. Approaches (*e*.*g*., small molecular preconditioning) that do not manipulate the genome of the transplanted stem cells should be the best way to provide safe stem cells for clinical applications.

Rho family GTPase signaling and its major downstream effector, Rho-associated-coiled-coil-forming protein kinases (ROCKI and ROCKII), involve diverse intracellular signal transduction pathways and regulate a wide range of fundamental cellular functions, such as cell proliferation, apoptosis, contraction, adhesion, and migration[13]. Y-27632 [(R)-(+)-trans-4-(1-Aminoethyl)-N-(4-pyridyl) cyclohexane carboxamide dihydrochloride] is a potent inhibitor of ROCK I and ROCK II[13, 14]. The initial application of Y-27632 in the stem cell field demonstrated its pronounced ability to protect dissociated single cells or cryopreserved human ESCs from apoptosis, improve human ESC survival, and enhance the efficiency of colony formation, thus, maintain self-renewal of human ESCs independent of animal-derived extracellular matrices[14]. Since then, numerous studies have found the same or similar protective effects on other cell types, such as human mesenchymal stem cells, corneal endothelial cells, and human ESC-derived cardiomyocytes[13, 15]. Y-27632 has even been used as a therapeutic drug to treat cardiovascular diseases[16].

Here, we hypothesize that Y-27632 can be used as a preconditioning reagent to protect human CSCs from apoptosis induced by Doxorubicin (Dox, brand name-Adriamycin). Apoptosis can be induced in many ways, including through chemicals (*e*.*g*., Dox, Puromycin, and Hydrogen peroxide), physical factors (*e*.*g*., UV, X-ray, and FBS-free starving culture), and/or biological molecules (*e*.*g*., TNF-α, Fasl, and TGFβ). In this study, Dox was used as the apoptotic inducer for two reasons: (1) Dox is one of the most effective and commonly used chemotherapeutic drugs to treat cancer patients, but, unfortunately, a notorious side-effect of Dox is its cardiotoxicity, which often results in cardiomyopathy and, eventually, congestive heart failure, and (2) Dox-induced cardiac toxicity is highly associated with apoptosis and necrosis within cardiomyocytes. Recently, studies suggested that Dox may impair and/or deplete endogenous cardiac stem cells, which may result in permanent damage to the heart[17]. Thus, our goal was to examine whether pretreatment with Y-27632 protected CSCs from apoptosis. We hope that the drug can be used to increase cell survival in stem cell therapy for heart disease or be used as a cardiac protective agent in cancer patients.

## Materials and Methods

### Experimental Material and Cell Culture

Cell culture wares were purchased from Fisher Scientific (Pittsburgh, PA) and/or USA Scientific (Ocala, FL). All vendor sources for specific equipment and reagents, such as microscopes, basal medium, FBS, growth factors, antibodies, chemical components, and assay kits are listed separately in each section below unless otherwise indicated.

Briefly, patient heart samples (atrial appendages) were obtained as discarded tissues from local hospitals. Donor confidentiality was maintained at the hospitals and no patient identification information or medical history was collected according to the approved protocol. A written consent agreement was obtained for collection of discarded atrial appendages by the hospitals and all procedures were approved by the Institutional Review Board (IRB) of Virginia Polytechnic Institute and State University for human subject research. The isolation and culture of human CSCs were previously described in the reference [18]. Briefly, the isolated cells were cultured in a 37°C incubator (5% CO_2_ and 21% O_2_) with CSC medium consisting of Ham’s F12 (Life Technology, Grand Island, NY), 10% FBS (JR Scientific, Woodland, CA), 10 ng/ml human bFGF (PeproTech, NJ), 0.2mM L-glutathione, and 0.005 U/ml human erythropoietin (both from Sigma-Aldrich, St. Louis, MO). After 3–5 day’s culture, with medium changes every other day, confluent cells were detached using TrypLE Express (Life Technologies) and reseeded at a density of 5x10^5^ per 100mm dish, 3x10^5^ per 60mm dish, 1.5x10^5^ per well in 6-well plates, 0.5x10^5^ per well in 12-well plates, or 3000 per well in 96-well plates followed by 1–2 days culture before drug treatment using Y-27632 (Fisher Scientific) and/or Dox (Sigma-Aldrich) at the concentrations described below.

The overall experiments include Y-27632 (0, 0.1, 1, 10μM), Dox (0, 0.2, 0.4, 0.6, 0.8, 1.0μM), and Y-27632 plus Dox groups, where 0 concentration of drug was used as control. The detailed information is addressed individually in each assay below.

### Cell Viability Assay Using Calcein-AM

After 2 days of culture in a 96-well plate, cells were treated with Y-27632 (0.0, 0.1, 1.0, and 10.0μM) or Doxorubicin (0.0, 0.2, 0.4, 0.6, 0.8, and 1.0μM) for 48hrs followed by Ham’s F12 wash and incubated with 2μM Calcein-AM (Life Technologies) for 30min in the dark at 37°C. Both fluorescent microscopy (Olympus IX 71, Center Valley, PA) and a Spectra Max plate reader (Molecular Device, Sunnyvale, CA) were used to obtain the original florescent images or the Relative Florescence Intensity (RFI) after staining. The Calcein positive cells (green) correspond to the live cells on the fluorescent images. RFI from the plate reader was used to calculate the ratios or fold changes of drug-treated groups over the control condition.

### Caspase-3 Activity Analysis

QuantiFluo Caspase-3 kit (BioAssay System, Hayward, CA) was used to measure Caspase-3 activity. Briefly, after 2 day treatments with Y-27632 (0.0, 0.1, 1.0, 10.0μM), Dox (0.0, 0.2, 0.4, 0.6, 0.8, 1μM), or Y-27632 (0.0, 0.1, 1.0, 10.0μM) plus Dox (0.6μM), both adherent and floating (dead) cells were collected. The cellular pellets were resuspended in 200μl lysis buffer (50mM HEPES, 100mM NaCl, 0.5% Triton X-100, pH7.2) on ice for 30min and the cell suspension was then rapidly frozen at -80°C until used. Protein content was assayed using Bradford reagents (Sigma-Aldrich). To measure Caspase-3 activity, aliquots of cell extracts containing 20μg total protein were incubated in a reaction buffer containing 20mM HEPES (pH7.4), 0.1% CHAPS, 5mM DTT, 2mM EDTA, and 20μM of the Caspase substrate N-acetyl-Asp-Glu-Val-Asp-7 amino-4-methylcoumarin (Ac-DEVD-AMC, BioAssay System) for 2hrs at room temperature (RT). The released AMC was measured as RFI using a 96-well Spectra Max fluorescence plate reader at Ex 360nm and Em 460nm.

### Apoptotic and Necrotic Assay with Annexin V and Propidium (PI)

CSCs cultured in 12-well plates were treated with 10μM Y-27632 or Y-27632 plus 0.6μM Dox for an additional 2 days. Both adherent and floating (dead) cells were collected and combined for the apoptotic and necrotic assays using the Annexin V kit (BioLegend, San Diego, CA). Briefly, cells were washed twice with cold PBS and incubated with 100ul Annexin V-FITC buffer (10mM HEPES, 150mM NaCl, 5mM KCl, 1mM MgCl_2_, 2.5mM CaCl_2_ supplemented with 5 μg/ml PI, 5 μg/ml Hoechst 33342 (Sigma-Aldrich) and with Annexin V-FITC diluted 1:40, pH7.4) in the dark for 15min at RT. After two washes with 1X binding buffer (10mM HEPES, 150mM NaCl, 5mM KCl, 1mM MgCl_2_, 2.5mM CaCl_2_, pH7.4), the cells were cytospun on slides using a Cytospin 4 centrifuge (Thermo Scientific, Waltham, MA). The fluorescence images were taken with an EVOS microscope (Life Technologies) or run with a BD FACSAIR flow cytometer (BD Biosciences, San Jose, CA). When Annexin V-FITC is used in conjunction with Propidium Iodide (PI), a vital dye, it is able to distinguish the apoptotic cells (Annexin V-FITC-positive, PI-negative) from necrotic/late-apoptotic (Annexin V-FITC-positive, PI-positive) cells[19].

### Immunocytochemical Staining Using C-kit Antibody

After a 4-day culture in the presence of 10μM Y-27632, cells were fixed with 4% PFA and followed by blocking in 1.5% BSA and 0.2% Tween in PBS at RT. The primary c-kit^+^ antibody at 1:100 (Cell Signaling Technology, Beverly, MA) was incubated at 4°C overnight followed by the secondary antibody (Alex Fluor 488 at 1:1000, Life Technologies) and Hoechst co-incubated at RT for 2hrs. The fluorescence images were obtained using an EVOS microscope. C-kit^+^ cells were calculated from the total number of nuclei stained with Hoechst. Approximately 100–200 nuclei were counted per image and 6 images per experiment were averaged for statistical analysis.

### Cell Proliferation Assay Using the EdU Kit

CSC proliferation was determined using the Click-iT EdU Alexa Fluor 488 Kit according to manufacturer’s protocol (Life Technologies). Briefly, at the end of a 2-day treatment with Y-27632 in 12-well pates, 10μM final concentration of EdU (5-ethynyl-2'-deoxyuridine) was added to the Control (0μM Y27632) or Treated (10μM Y-27632) wells in triplicate. After an additional 16hrs culture in the 37°C incubator, the cells were washed with warm PBS and fixed/permeabilized with 4% PFA and 0.5% Triton X-100 for 20min at RT. The cells were continuously kept in the dark for 30min with 250μl of Click-iT reaction mixture (1X Click-iT reaction buffer, CuSO_4_, Alexa Fluor 488-azide, and reaction buffer additive). After being washed with 3% BSA and PBS, nuclei were counterstained with DAPI (4,6-diamidino-2-phenyli, Fisher Scientific) and the fluorescent images were taken using an EVOS fluorescence microscope. The % EdU positive cells were calculated among the total number of nuclei stained with DAPI. Approximately 200 nuclei were counted per image and five images per experiment were averaged for statistical analysis.

### Western Blot

CSCs cultured in 60-mm dishes were lysed by scraping in cold RIPA (50mM Tris-HCl, 150mM NaCl, 1% IGEPAL-CA630, 0.5% sodium deoxycholate, 0.1% SDS, pH 8.0, from Sigma-Aldrich) containing freshly added Halt Phosphatase Inhibitor Cocktail (Fisher Scientific) and Protease Inhibitor Cocktail (Sigma-Aldrich) on ice for 45min. Electrophoresis (20μg of total protein per sample) was carried out in a 10% or 12% SDS-PAGE gel using an electrophoresis system (Bio-Rad, Hercules, CA) and proteins were blotted onto PVDF membranes. The membranes were then blocked for 1hr at RT using 5% blocking solution before the primary antibodies (β-actin at 1:1000, Caspase-3 at 1:200, Bcl-xl at 1:1000, Bcl2 at 1:1000, or Bax at 1:1000 ratio, all from Cell Signaling Technology, Beverly, MA) were incubated at 4°C overnight followed by HRP-conjugated secondary antibodies (Jackson Immuno Research laboratories, West Grove, PA) for 2hrs at RT. The chemiluminescence signals were generated using Clarity ECL Western Blotting Substrate (Bio-Rad) and detected on a Kodak Image Station 4000 equipped with Carestream MI v5.3 software.

### In Vitro Wound Healing Assay

CSCs were cultured in 12-well plates to 80–90% confluence. The *in vitro* “Wound Healing” was created by slightly scraping the confluence culture along the middle line of a well using a 10 μL plastic pipette tip[15]. The scraped cell suspension was washed with PBS and replaced with fresh CSC culture medium containing 0μM or 10μM Y-27632 in addition to 10μM EdU. The “wound” size (*i*.*e*., the distance crossing two edges of the scraped line) was determined using ImageJ on images taken at 0, 2, 4, 6, 8, 10, 12, and 24hrs after treatment. Cell proliferation was also assessed by the % EdU positive cells and the number of DAPI positive nuclei per region of interest as described previously[15].

### Statistical Analysis

All data are presented as Mean ± SEM (Standard Error of Mean) unless otherwise stated. Student’s t test with a two-tailed distribution was used to compare two groups. One-way ANOVA followed by the Bonferroni test was used to compare three or more groups with *p<0*.*05* being considered statistically significant. GraphPad Prism 5 and Microsoft Excel 2010 were used for statistical analysis and plotting.

## Results

### Toxic Effects of Dox on Cardiac Stem Cells

**Fig 1:**
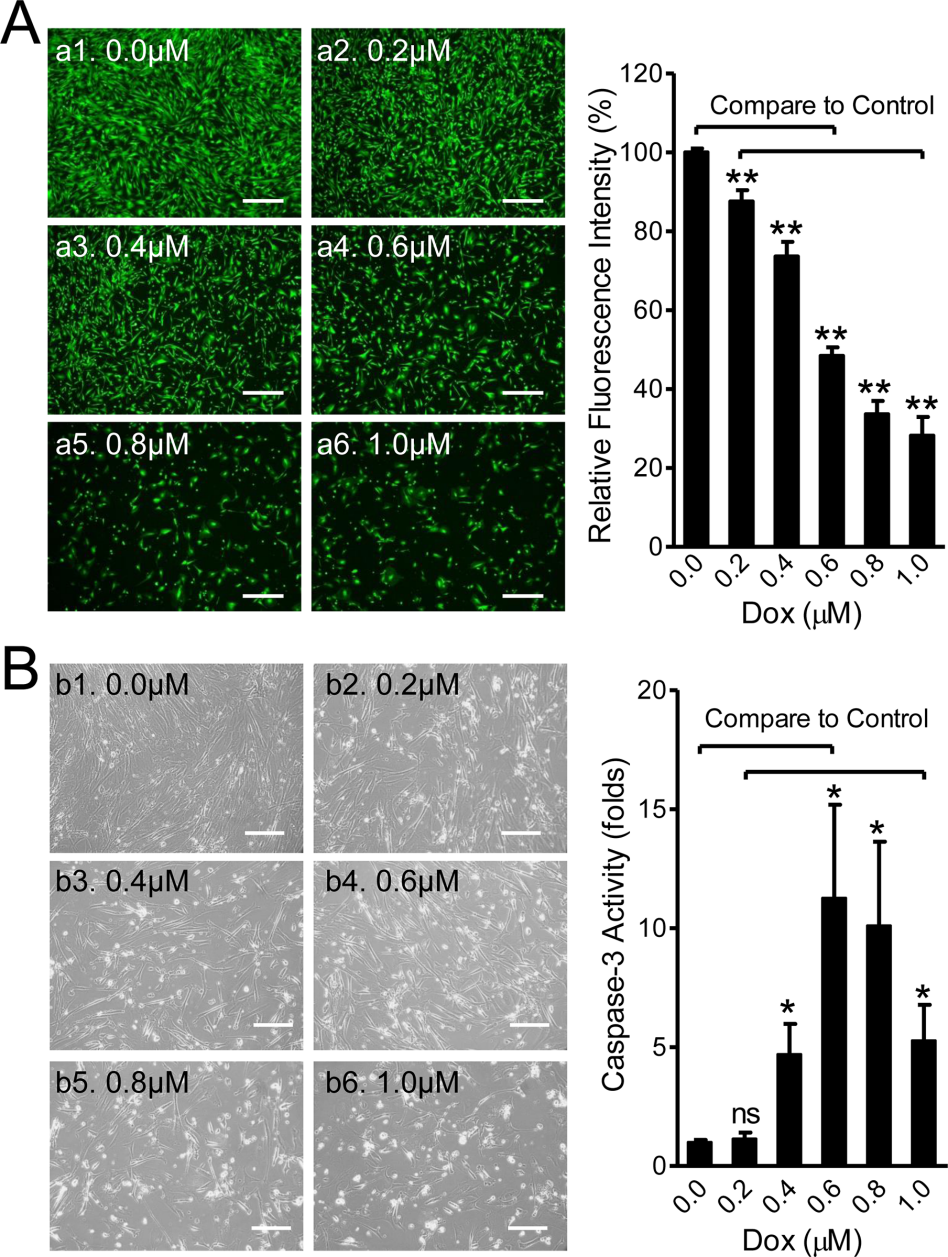
Doxorubicin (Dox)-induced Apoptosis in CSCs via Increased Caspase-3 Activity. **(A)** Cell viability under Dox-treatment. CSCs were treated with Dox for 48hrs at the indicated concentrations. Fluorescent microscopy images represent live cells (in green) stained with Calcein-AM. Dead floating cells were washed away during medium changes. Scale bar = 500μm. The means of viability rates (%), calculated by normalizing fluorescent intensities of Dox-treated groups to control levels, are shown in the bar graphs on the right. The % of viable cells are inversely proportional to the concentrations of Dox (n = 9; **: *p*<0.01). **(B)** Caspase-3 activity under Dox-induced apoptosis. During 48hrs treatment, more dead cells (white spots) were observed and live cell density was gradually reduced as Dox concentrations increased (b1 to b6). Scale bar = 200μm. Means of Caspase-3 activities are shown in the bar graph on the right with a maximum response at 0.6μM Dox (n = 9; ns: no significance, *: *p*<0.05).

Dox-induced cardiotoxicity and cardiomyopathy are believed to be involved in Dox-induced apoptosis of cardiomyocytes and/or cardiac progenitor cells[17]. To determine whether Dox causes similar toxic effects on *in vitro* human CSCs, cells were exposed to 0, 0.2, 0.4, 0.6, 0.8, and 1.0μM of Dox for 2 days. Calcein-AM staining and Caspase-3 assays were used to evaluate cell viability and apoptosis, respectively. As shown in Fig 1A, numbers of viable cells and means of fluorescence intensities were significantly decreased in a dose-dependent manner by Dox (n = 9, *p<*0.01 at all concentrations). At 0.6μM Dox, about 50% (48.43±2.2%) of the cells were viable, while only ~30% of the cells were able to survive when treated with Dox at 1μM. Morphologically, cells were found to show membrane blebbing at about 12hrs after treatment, followed by round-up and detachment. Dead cells were usually floating in the supernatant and could be washed away during medium changes.

To further examine whether Dox-induced apoptosis involves the Caspase pathway, Caspase-3 (the last component of both the extrinsic and intrinsic apoptotic cascade) activity was measured with the QuantiFluo Caspase-3 kit. It was found that Dox-treatment induced significant cell death (the white spots, Fig 1B, the left panel) and the number of dead cells seemed to be in proportion to Dox concentration. When total cell extracts collected from both adherent viable cells and floating dead cells were used, a dramatic increase in Caspase-3 activity in a dose-dependent manner was observed. At 0.6μM Dox, Caspase-3 activity reached its maximum level (11.3±3.9 fold higher) and then gradually decreased at higher Dox concentrations (Fig 1B, the right panel).These results indicate that Dox-induced apoptosis in CSCs likely involves Caspase-3 dependent pathways, which is commonly reported in other cell types such as cardiomyocytes and mesenchymal stem cells[17, 20]. Since 0.6μM Dox resulted in ~50% dead cells and the maximal increase in Caspase-3 activity, this concentration was used in subsequent experiments unless otherwise stated.

### Potential Beneficial Effects of Y-27632 on Cardiac Stem Cells

**Fig 2:**
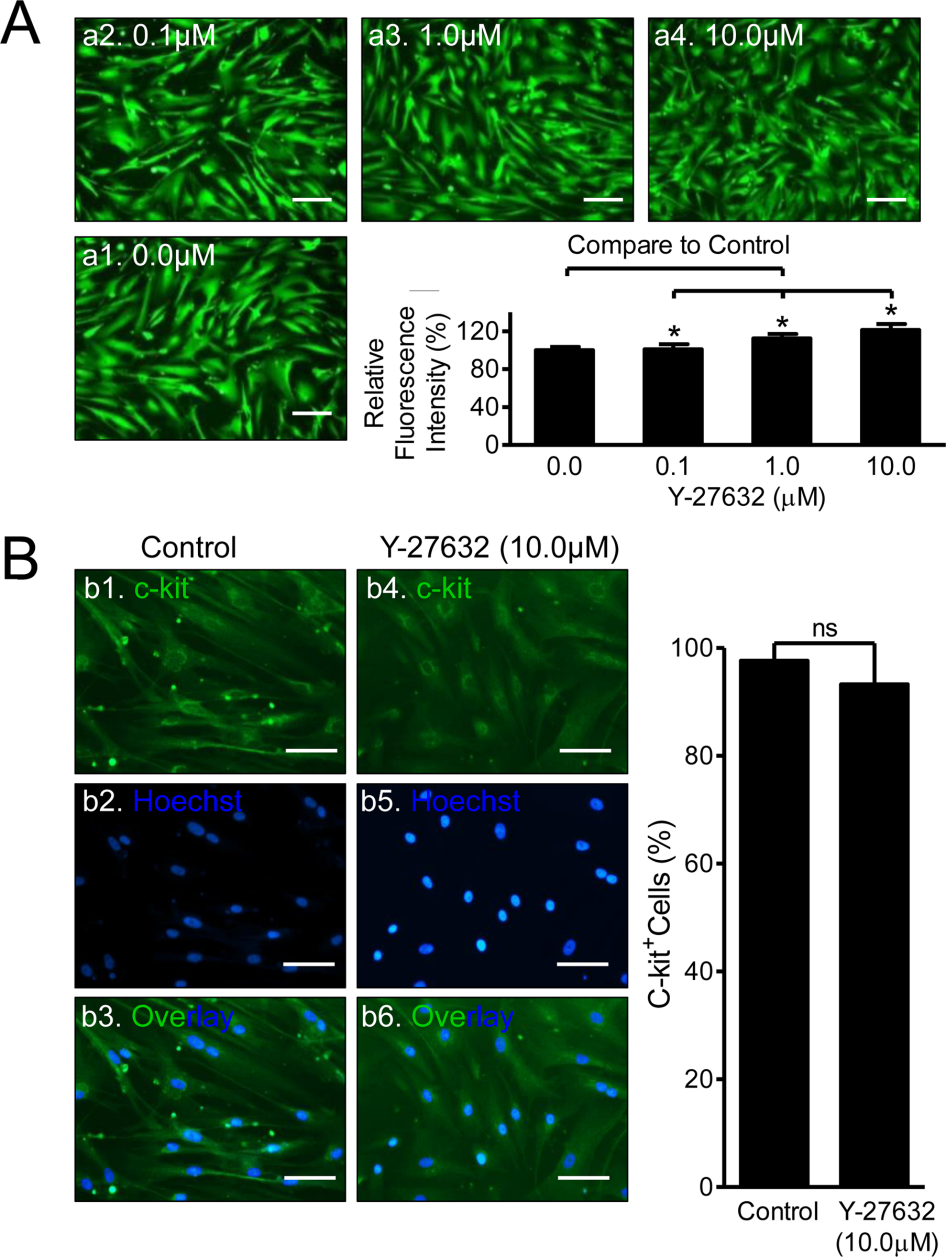
Effects of Y-27632 on Viability and c-kit^+^ Expression of CSCs. **(A)** Cell viability assay using Calcein-AM. CSCs were treated with Y-27632 (0.0, 0.1, 1.0, & 10.0μM) for 48hrs followed by Calcein-AM staining. Representative images (a1 to a4) of fluorescent microscopy display the live cells (in green). Scale bar = 200μm. Relative cell viability (%) was analyzed by normalizing the means of fluorescent intensity to the control level as 100%, shown in the bar graphs on the right. *: *p*<0.05, n = 12. **(B)** The c-kit^+^ expression of CSC in Control and 48hrs after 10μM Y-27632 treatment. Adherent CSCs were stained with anti-human c-kit^+^ specific antibody followed by the secondary antibody-conjugated with FITC. C-kit^+^ CSCs are shown in green (b1, b3, b4, & b6) and nuclei are in blue (b2, b3, b5, & b6). Scale bar = 100μm. Percentages of c-kit^+^ CSCs from both Control and Y-37632-treated groups are shown in the bar graphs on the right. No significance was observed between the two groups (ns: no significance, n = 3).

The ROCK inhibitor, Y-27632, demonstrates anti-apoptotic benefits in ESCs and iPSCs[14]. To examine whether Y-27632 had any effect on CSC viability or changes stem cell properties (c-kit^+^ expression), cells were cultured in 96-well plates for 2 days followed by treatment of Y-27632 (0, 0.1, 1, and 10μM in triplicate) for an additional 2 days. The representative Calcein-fluorescent images of viable cells show increases in cell density, as the concentrations of Y-27632 increased to 1 and 10μM (Fig 2A). Quantitative analysis using the means of fluorescence intensity displayed significant increases in RFI in a dose-dependent manner compared with Control (Fig 2A, bar graphs, n = 12, *p<0*.*05*). Importantly, Y-27632 treatment did not change c-kit^+^ expression level, as indicated by the high levels of c-kit^+^ CSCs in both Control (96.9±0.4%) and Y-27632 (95.0±1.0%, n = 3, ns) groups shown in Fig 2B. These data suggest that ROCK inhibition increased CSC viability without changing stem cell properties.

**Fig 3:**
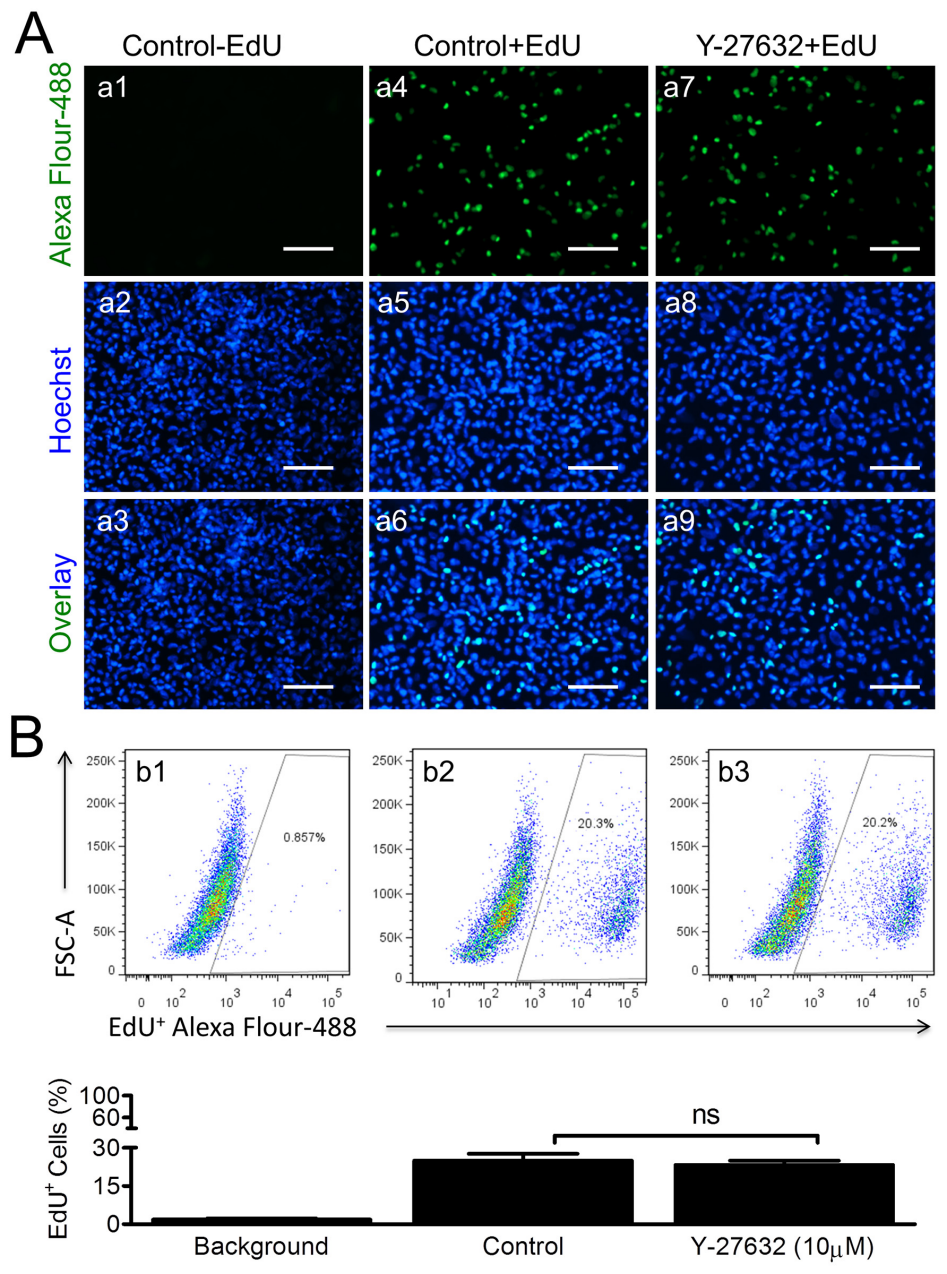
No Significant Effects of Y-27632 on Cell Proliferation. **(A)** Representative fluorescent images of cell proliferation evaluated with the EdU (5-ethynyl-2'-deoxyuridine) kit. EdU^+^ proliferative cells are shown in green (a4, a6, a7, & a9) and the corresponding nuclei were stained in blue (a5, a6, a8, & a9). No EdU positive cells were found in the Control-EdU group (a1 to a3). Scale bar = 200μm. **(B)** Display of the proliferation rates (%) measured with Flow Cytometry. Means of 3 separate measurements are plotted in the bar graphs below (n = 3, ns: no significance). No statistical significance was found between the Control+EdU and the Y-27632+EdU groups.

To determine whether the increased RFI in Fig 2A was also due to rising cell proliferation under Y-27632 treatment, we next performed cell proliferation assays using the EdU-Alexa Fluor 488 kit in combination with a Fluorescent Microscope and Flow Cytometer. Cells were cultured in 6-well plates for 2 days in the absence or presence of 10μM Y-27632, a common concentration used for ESCs/ iPSCs[13, 14]. Both fluorescence images (Fig 3A) and Flow Cytometer (Fig 3B) analysis did not result in statistical significance of EdU positive (*i*.*e*., proliferative) cells between Control and Treated groups. The means of EdU^+^ cells counted in fluorescent images were 24.54±1.44% (Control + EdU, 319/1300 cells) and 21.23±4.4% (Y-27632 + EdU, 238/1129 cells) (n = 12, ns, data not shown in Fig 3A). Flow Cytometry assays showed the comparable percentages of EdU^+^ cells between Control (24.9±2.8%) and Treated groups (23.3±1.6%) (Fig 3B, n = 3, ns). Although the percentage of EdU^+^ cells measured by Flow Cytometry was marginally lower than those counted from fluorescent microscopy images, the % difference was not significant. Taken together, these data suggest that Y-27632 treatment had no effect on CSC proliferation, but did enhance cell viability.

**Fig 4:**
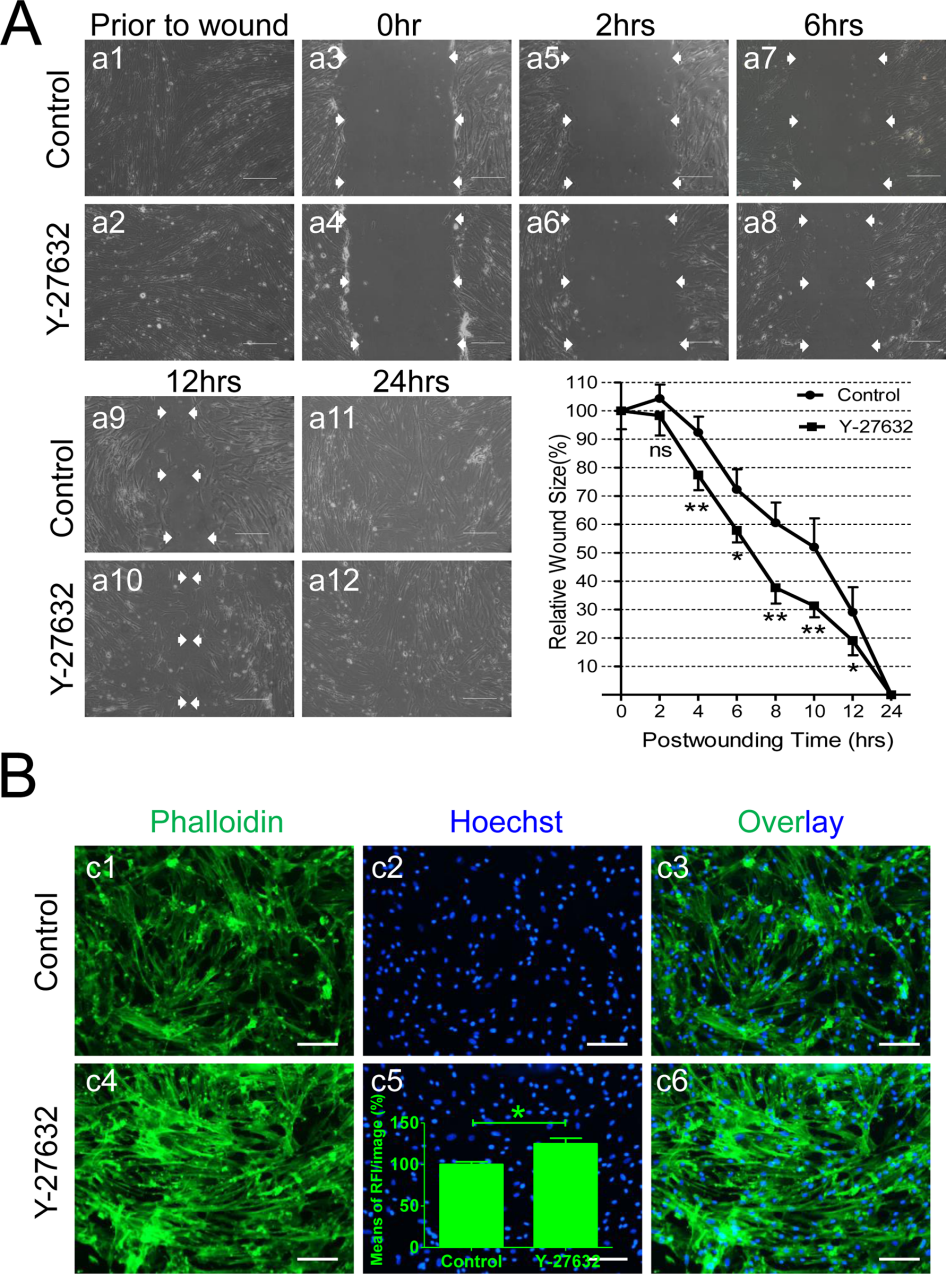
Y-27632 Enhances Cell Migration in Wound Healing Assay. **(A)** Representative phase-contrast microscopy images of CSC migration and time-course of wound healing after 10μM Y-27632 treatment for 48hrs. The “Wound Sizes” were measured by determining the means of at least 3 points along each edge as indicated with white arrows (a3 to a10). Scale bar = 500μm. The relative “wound sizes (%)” of Control (filled circle) and Y-27632-treated (filled square) cells were compared at 0, 2, 6, 12, & 24hrs post-wounding by normalizing to 0hr. ns: no significance, *: *p*<0.05, **: *p*<0.01, n = 6. **(B)** Phalloidin staining to evaluate formation of cellular F-actin filaments after treatment with 10μM Y-27632 for 48hrs. The representative fluorescent microscopy images display F-actin in green (Phalloidin) and nuclei in blue (Hoechst). Scale bar = 200μm. The means of relative fluorescence intensities per image were analyzed and plotted as an insert in Fig B, c5 (*: *p*<0.05, n = 5).

Published studies report that ROCK inhibition enhances cell adhesion and wound healing in human corneal endothelial cells[15]. To determine whether Y-27632 incubation affects human CSC motility, which may be the cause of increased cell viability (above) or enhanced cell migration (below), we employed an *in vitro* “wound healing” model for this test[15]. Compared to Control groups, Y-27632-treated-group displayed significantly enhanced wound healing at about 4hrs and wounds were completely closed after 24hrs (*p<*0.05 or 0.01 between 4hrs and 12hrs, n = 6 for all groups in Fig 4A). It took ~6.5hrs in the Y-27632 treated groups to reach 50% wound closure versus ~10hrs in Control groups (Fig 4A, the low right panel). Additional EdU tests (data not shown) in the wound healing assay excluded the possibility that enhanced cell migration was due to cell proliferation, indicating that Y-27632-accelerated wound healing does not involve cell proliferation.

One of the major cytoskeletal proteins, Actin, is known to play important roles in many cell functions, such as wound healing and apoptosis[21]. It is known that actin is present in two forms. One is a free globular monomer called G-actin and the other is a double-stranded filamentous polymer called F-actin and these two forms can be regulated by many intracellular signal transduction pathways, including ROCK I[21]. Thus, inhibition of the ROCK system in human CSCs likely changes cellular mobility, division, contraction, and apoptosis. The representative fluorescent images in Fig 4B demonstrated that Y-27632 significantly increased the content of F-actin stained with Phalloidin[22](indicated by the green fluorescent intensities), compared with Control. Quantitative analysis of mean fluorescent intensities using Image J resulted in a significantly higher RFI in Y-27632 (124.8±6.7RFI) compared to Control (100.0±3.1RFI, *p<0*.*01*, n = 5, Fig 4B, inserted bar graph), indicating that the Y-27632-induced wound healing and cell migration probably occurred by facilitating formation of F-actin from G-actin[13, 14, 23].

### Preconditioning of Y-27632 Attenuated Dox-induced Apoptosis in Cardiac Stem Cells

Not only does the ROCK inhibitor (Y-27632) protect the dissociated or cryopreserved stem cell from apoptosis as mentioned above[14], a recent gene (ROCK1) knock-out study in mouse embryonic fibroblasts demonstrated its remarkable anti-apoptotic, anti-detachment, and pro-survival effects against apoptosis induced by Dox[24], suggesting a potential common feature of the ROCK-mediated apoptotic pathway in other cells types, including human CSCs.

**Fig 5:**
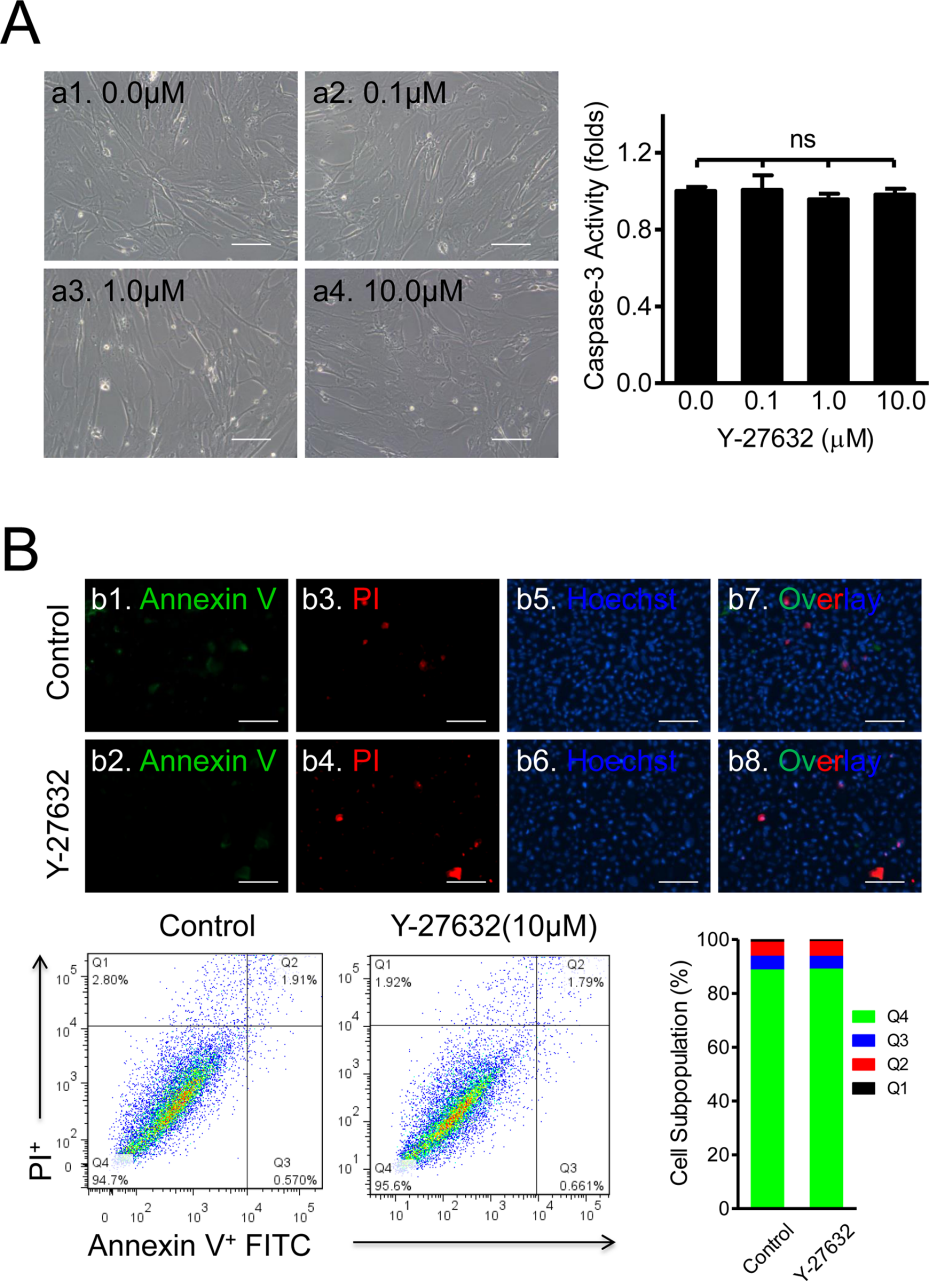
Y-27632 Treatment has no Effect on Apoptosis and Necrosis in CSCs under Basal Conditions. **(A)** No dose-dependent effect of Y-27632 on Caspase-3 activity under basal conditions. CSCs were treated with Y-27632 at the indicated concentrations for 48hrs. The representative phase contrast microscopy images are shown on the left. Scale bar = 100μm. Quantitative analysis using the Caspase-3 kit did not generate statistically significant differences between Control and Y-27632-treated groups (n = 15; ns: no significance). **(B)** Fluorescent microscope images of cell apoptosis and necrosis stained with Annexin V/PI. After treatment with 10μM Y-27632 for 48hrs, all cells (floating and adherent) were collected and stained with Annexin V-FITC/PI/Hoechst. The representative images show very few apoptotic cells (in green) and necrotic cells (in red) among many nuclei (in blue). Scale bar = 100μm. The corresponding flow data illustrates the necrotic cells (Q1), necrotic or late-apoptotic cells (Q2), early apoptotic cells (Q3), and normal cells (Q4), respectively. The distribution of subpopulations is shown in the bar graph on the right. No statistically significant differences for apoptotic and necrotic cells were found between Control and Treated groups (n = 3).

**Fig 6:**
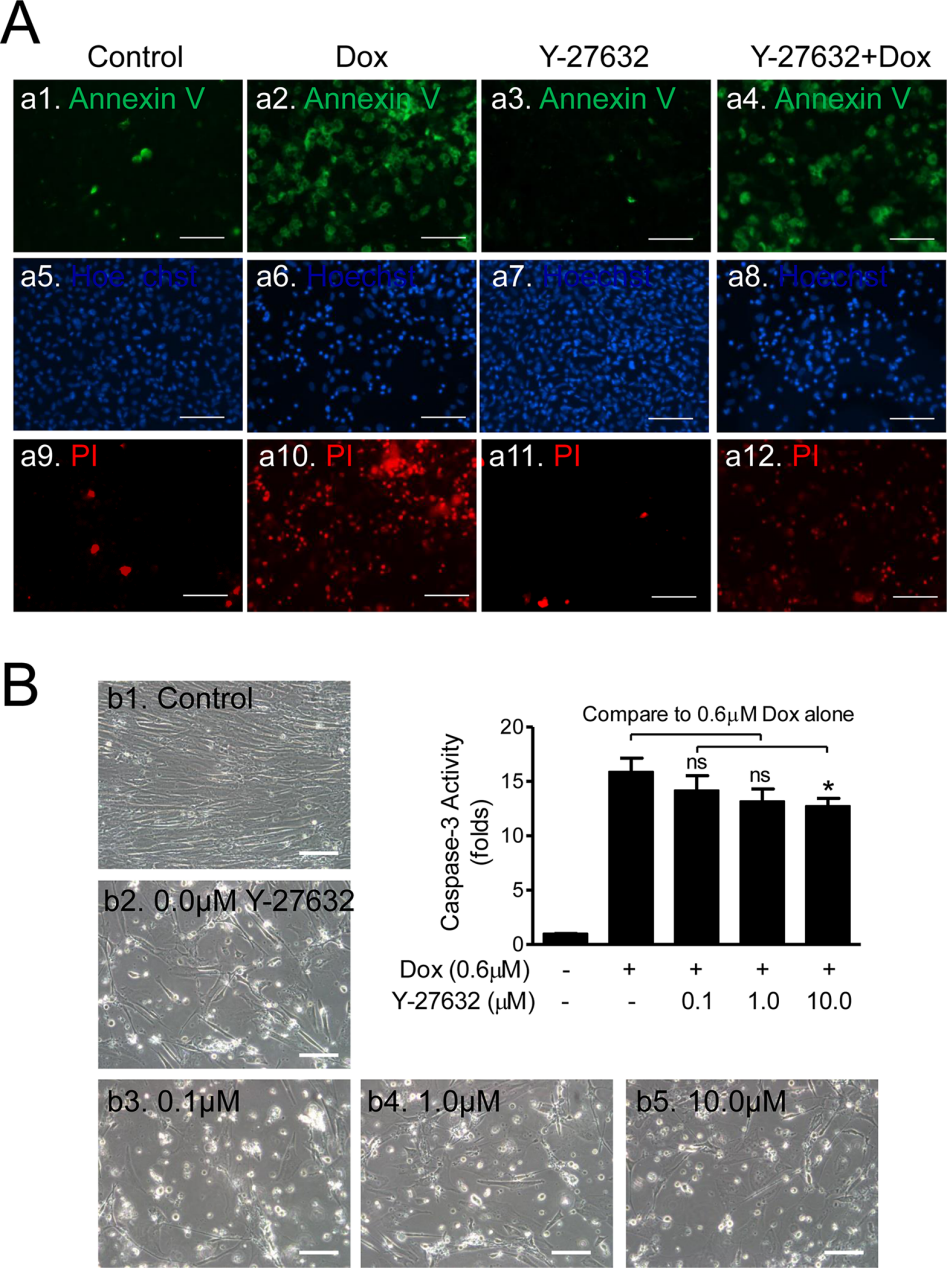
Pretreatment with Y-26732 Decreased Caspase-3 Activity in CSCs Induced by Dox. **(A)** Fluorescent microscope images of apoptotic/necrotic cells stained with Annexin V/PI kit. CSCs were pretreated with 10μM Y-27632 for 48hrs followed by 48hrs exposure to 0.6μM Dox. Annexin V (a1 to a4, in green), Hoechst (a5 to a8, in blue), and PI (a9 to a12, in red) fluorescent images show the reduced apoptotic (a4) and necrotic (a12) cells compared with Controls (a2 and a10), respectively. Scale bar = 100μm. **(B)** Effect of pretreatment with Y-27632 on apoptosis induced by Dox. CSCs were pretreated with various concentrations (0.0, 0.1, 1.0, & 10.0μM) of Y-27632 for 48hrs followed by 48hrs exposure to 0.6μM Dox. The representative phase contrast microscope images were taken at the end of each treatment. Control (b1) refers to “no Y-27632, no Dox” and the rest of the images (b2 to b5) indicate the tested concentrations of Y-27632 plus 0.6μM Dox. Scale bar = 100μm. The Caspase-3 kit was used to determine the Caspase-3 activity in the plate reader and the means of the fold changes are shown in the bar graphs in the upper right hand corner (n = 18; ns: no significance, *: *p*<0.05).

First, we determined whether Y-27632 downregulates Caspase-3 at basal conditions (no Dox). CSCs were treated with Y-27632 at the concentrations of 0.0, 0.1, 1.0, and 10.0μM for 2 days before the Caspase-3 activities were measured with QuantiFluo Caspase-3 kit. The phase-contrast images of the Control and Treated groups showed no notable differences in cell morphology and cell health status (Fig 5A, the left panel). The means of Caspase-3 activity showed no statistically significant differences between Control (1±0.02 fold) and the Y-27632 group (1±0.08 fold at 0.1μM, 0.96±0.03 fold at 1μM, 0.98±0.03 fold at 10μM, n = 15, ns) (Fig 5A, the right panel). Further, fluorescent images (Fig 5B, the top panel) and Flow Cytometer analysis (Fig 5B, the bottom panel) using the Annex VI and PI kits confirmed these findings by showing no significant differences between Control and Y-27632, respectively, in Q1 (0.98±0.14% vs 0.71±0.14%), Q2 (5.22±1.5% vs 5.5±1.9%), Q3 (5.1±1.4% vs 4.7±1.2%), or Q4 (88.7±2.9% vs 89.1±3.1%) (Fig 5B, n = 3, ns for all comparisons). In contrast, when cells were subjected to apoptosis induced by Dox, Y-27632 pretreatment significantly attenuated the number of apoptotic and necrotic cells (Fig 6A). In comparison with Dox treatments (Fig 6A, a2, and a10), Y-276342 significantly decreased apoptotic (Annexin V positive in red) and necrotic cells (PI positive cells in green) (Fig 6A, a4 and a12).

To examine whether the protective effects of Y-27632 involved the inhibition of Caspase-3 in the apoptotic cascade, CSCs were pretreated with Y-27632 at various concentrations (0.0, 0.1, 1.0, or 10.0μM) followed by 0.6μM Dox. Cellular extracts from both adherent and floating cells were collected and Caspase-3 activities were measured using the QuantiFluo Caspase-3 kit. The phase contrast microscope images showed significant cell death (white spots, Fig 6B, b2-b5) under Dox-treatment, but the number of dead cells seemed in reverse proportion to the concentration of Y-27632 (*e*.*g*., b3 vs b5). On the other hand, the same concentration of Dox significantly increased Caspase-3 activity up to ~16 fold (15.88±1.25 fold) from the basal level (1±0.01 fold). However, the increased activity of Caspase-3 gradually decreased as the concentration of Y-27632 went up. For example, comparing “0.6μM Dox Only” (15.9±1.3 fold) to “0.6μM Dox Only + 10μM Y-27632” (12.7±0.7 fold), Caspase-3 activity was significantly reduced by about 20% by Y-27632 treatment (Fig 6B, *p<0*.*05*, n = 36). These data suggest that Y-27632-promoted anti-apoptotic effects may be through direct or indirect inhibition of Caspase-3.

**Fig 7:**
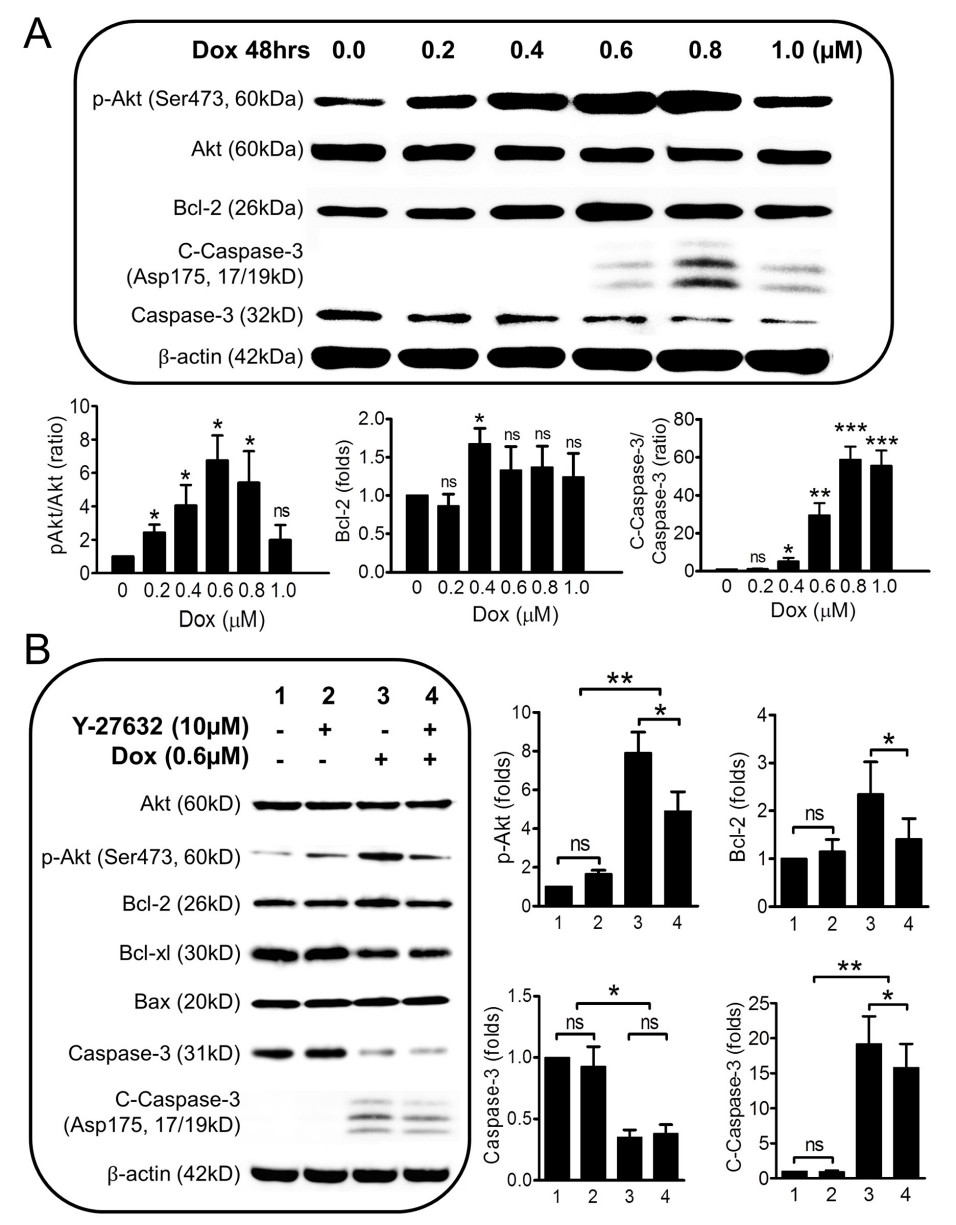
Effects of Dox alone and Y-27632 Plus Dox on Key Apoptotic Factors in CSCs. **(A)** The dose-dependent expressions of Akt/p-Akt/Bcl-2/Caspase-3/C-Caspase-3 in CSCs under Dox treatments. Briefly, CSCs were incubated with Dox at 0.0, 0.2, 0.4, 0.6, 0.8, and 1.0μM for 48hrs followed by Western blot analysis. The means of fold changes by Western blot shows a “Gaussian-type” response for pAkt/Akt and Bcl-2 but a “Sigmoid-type” for C-Caspase-3 with increasing concentrations of Dox. n = 3-4.ns: no significance; *: *p*<0.05; **: *p*<0.01; ***: *p*<0.001 compared to the basal condition (no Dox). **(B)** Pretreatment of Y-26732 significantly decreased the activity of cleaved Caspase-3 induced by Dox-treatment. In the experiment, CSCs were incubated with or without Y-27632 (10μM) for 48hr followed by Dox (0.6μM) for 48 hrs. Western blot was performed at the end of the treatments. The means of fold changes are shown in the right bar graphs except for those (Akt, Bcl-xl, and Bax) without statistical significance. For all panels, n = 3–6; ns: no significance; *: *p*<0.05; **: *p*<0.01; **Dox**: Doxorubicin; **Akt**: protein Kinase-B (PKB); **p-Akt:** phospho-Akt; **Bcl-2**: B-cell lymphoma 2; **Bcl-xl:** Bcl-extra large (or Bcl-2-like 1 isoform 1); **Bax**: Bcl-2-associated X protein; **C-Caspase-3**: cleaved Caspase-3.

To further explore the underlying mechanisms involving the protective effects of Y-27632, we used Western Blot to analyze the protein expression levels of several key factors in the apoptotic pathways. Our results showed: (1) Treatment with Dox (0.0, 0.2, 0.4, 0.6, 0.8, and 1.0μM) alone generated a “Gaussian-type” response of protein expressions for p-Akt/Akt (the peak values: 6.8±1.5 fold) and Bcl-2 (the peak values: 1.7±0.2 folds), but a “Sigmoid-type” response for cleaved Caspase-3/Caspase-3 (the peak value: 58.8±6.9 fold) at 0.4 to 0.6μM of Dox (Fig 7A, ns: no significance; *: *p*<0.05; **: *p*<0.01; ***: *p*<0.001 compared to basal condition, n = 3–4). In other words, the low concentrations of Dox (0.0 to 0.6μM) seemed to increase the expression of p-Akt and Bcl-2, but the high concentrations of Dox (0.6 to 1.0μM) decreased the expression of those factors (*i*.*e*., Gaussian-type response). Interestingly, the expression level of cleaved Caspase-3 was in direct ratio to Dox concentrations (*i*.*e*., Sigmoid-type response). (2) In contrast, treatment with 10μM Y-27632 alone did not result in notable changes in protein expression levels for ROCK I & II (ROCK family), Akt/p-Akt/Bcl-2/Bcl-xl (anti-apoptotic factors), and Bax/Caspase-3 (pro-apoptotic factors) (Fig 7B, not all data shown, n = 3–5, ns for all comparisons). Together, these data suggest that the anti-apoptotic effects of Y-27632 may not become “visible” unless cells are undergoing stress.

When CSCs were first pretreated with 10μM Y-27632 followed by 0.6μM Dox, complex expression patterns of the above factors were observed. As shown in Fig 7B, 0.6μM Dox alone (Lane 3) significantly increased p-Akt, (7.9±1.1 fold, *p<0*.*05*, n = 4), Bcl-2 (2.3±0.7 fold, *p<0*.*05*, n = 6), and cleaved Caspase-3 (19.2±4.0 fold, *p<0*.*05*, n = 6), but decreased total Caspase-3 (0.4±0.1 fold, *p<0*.*05*, n = 6) compared to Controls (Lane 1). Once cells were pretreated with Y-27632 (Lane 4), the following addition of Dox (Lane 4) would further increase Akt, but decrease p-Akt, Bcl-2, and cleaved Caspase-3. However, compared to Dox alone (Lane 3), Y-27632 + Dox (Lane 4) changed Akt levels from 0.82±0.1 fold to 0.9±0.1 fold (*p<0*.*05*, n = 4), p-Akt from 7.9±1.1 fold to 4.9±1.0 fold (*p<0*.*05*, n = 4), Bcl-2 from 2.4±0.7 fold to 1.4±0.4 fold (*p<0*.*05*, n = 6), and cleaved Caspase-3 from 19.2±4.0 fold to 15.8±3.4 fold (*p<0*.*05*, n = 6). In other words, Y-27632 did not result in notable changes in the key apoptotic factors listed above while Dox treatment produced more complex patterns of expression, consisting of an increase in p-Akt, Bcl-2, cleaved Caspase-3, a decrease in total Akt, total Caspase-3, and/or no changes in Bcl-xl and Bax. Once cells were preconditioned with Y-27632, Dox addition could not increase p-Akt, Bcl-2, or cleaved Caspase-3 to the same levels as it did without Y-27632, indicating that: (1) Y-27632 protects cells likely by inhibiting Dox-induced upregulation of cleaved Caspase-3, possibly not through Akt/Bcl-2 pathway and (2) The increased expression of p-Akt and Bcl-2 after Dox treatment may demonstrate the successful development of a drug resistant system (a common protective machinery in cancer cells against chemotherapy, see Discussion below) in cardiac stem cells.

## Discussion

**Fig 8:**
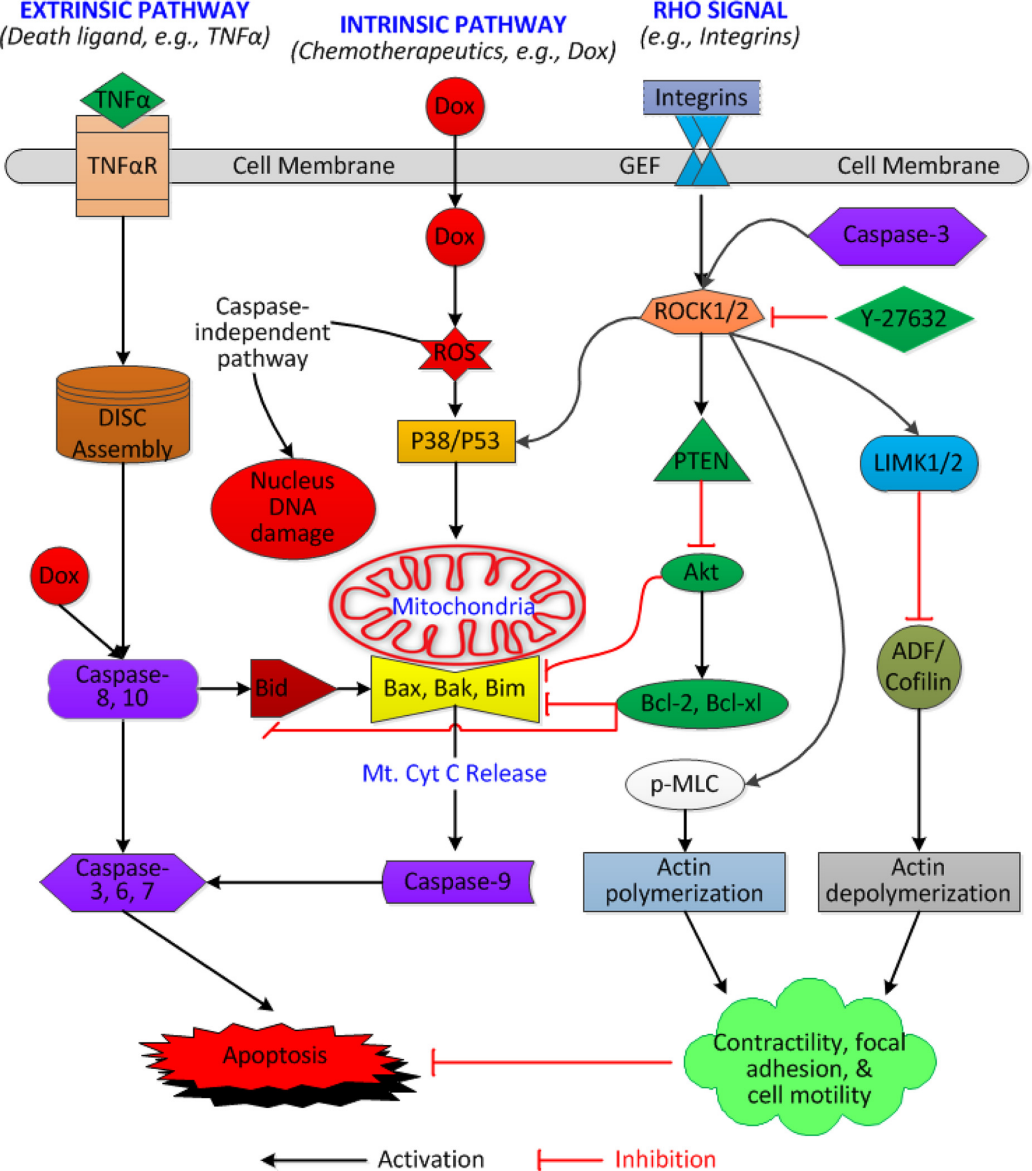
A Working Hypothesis of ROCK Inhibition-induced Anti-apoptotic Mechanism in Human CSCs under Dox-treatment. The apoptotic pathways are commonly agreed to consist of the extrinsic and intrinsic pathways[25]. Chemotherapeutic drug (*e*.*g*., Dox)-induced apoptosis likely involves both pathways and a Caspase-independent route[26]. Inhibition of ROCK I and II result in the attenuation of Dox-induced apoptosis. Notes: **ADF/Cofilin**: actin depolymerizing factor Cofilin protein; **Bak:** Bcl-2 homologous antagonist/killer; **Bid:** BH3 interacting-domain death agonist; **Bim:** Bcl-2 interacting mediator of cell death; **Cyt C:** Cytochrome C; **DISC:** death-inducing signaling complex; **GEF:** Rho guanine nucleotide exchange factor; **LIMK1/2:** LIM kinase-1 & -2; **p-MLC**: phosphorylated myosin light chain; **Mt:** Mitochondria; **PTEN:** Phosphatase and tension homologue; **ROS:** Reactive oxygen species; **TNFαR:** Tumor necrosis factor-α receptor; For other abbreviations, please see Fig 7 and the main text.

The present study demonstrates that Dox alone significantly increased Caspase-3 activity and promoted apoptosis of human CSCs in a dose-dependent manner. Although Y-27632 alone did not result in the observed changes in apoptosis, stemness (c-kit expression), cell proliferation, and protein expression levels of the above factors, it greatly enhanced cell viability, F-actin formation, and cell migration in the wound healing assay. Once cells were pretreated with Y-27632, Dox-induced expression of cleaved Caspase-3 and cell death was dramatically reduced. Based on these findings, we propose a working hypothesis of a ROCK inhibition-induced anti-apoptotic mechanism, which may involve the ROCK to Caspase-3 and/or ROCK to F-actin pathways (Fig 8). To the best of our knowledge, this is the first study to demonstrate the protective effects of Y-27632 on human CSCs under Dox-induced apoptosis.

Since its biological importance was first reported in smooth muscle cells in 1997[27], Y-27632 has been demonstrated to play crucial roles in numerous physiological functions, such as cell proliferation, differentiation, adhesion, migration, inflammation, and/or apoptosis in various cell types, including ESCs/iPSCs[14], hematopoietic progenitor cells[28], epithelial stem cells, mesenchymal stem cells[29], stem cell-derived cardiomyocytes[30], tumor cells[31], and corneal endothelial cells[32]. Thus, the broad biological effects of Y-27632 have led to its new potential role as a therapeutic drug to treat various diseases, such as cardiovascular diseases[16].

Dox is well known as an effective chemotherapeutic agent to treat cancer patients, but one of its major side effects is cardiotoxicity, resulting in the direct cell death of cardiomyocytes and/or depletion of endogenous CSCs[17]. Dox-induced responses for apoptotic factors (especially anti-apoptotic ones) seemed to depend on the cell type tested. For differentiated cell types (*e*.*g*., cardiomyocytes[33] and endothelial cells[34]), Dox treatment usually resulted in the upregulation of pro-apoptotic factors (*e*.*g*., Bax, Bak, Bim, Bid, and/or Caspases) and downregulation of anti-apoptotic factors (*e*.*g*., Akt, Bcl-2, and/or Bcl-xl). In contrast, for stem cells (*e*.*g*., cardiac progenitor cells[35]) or tumor cells (*e*.*g*., human myeloma cell lines[36] and T-cell leukemia cell line[37]), Dox often produced the opposite results, *i*.*e*., it upregulated anti-apoptotic factors, but may not have effects on pro-apoptotic factors.

Our results showed that under basal conditions (no Dox), Y-27632 alone did not produce apparent effects on apoptosis/necrosis, CSC markers (c-kit), cell proliferation, or the protein expression of ROCKI/II and apoptotic-related key factors *(e*.*g*., Akt, p-Akt, Bcl-2, Bcl-xl, Bax, cleaved Caspase-3, and Caspase-3); however, Y-27632 did significantly increase cell viability, F-actin formation, and cell migration. These results are consistent with some published results [38] but not others [39], probably due to different cell types and experimental conditions. As to cell mobility, our findings are consistent with many reported results, where ROCK inhibition significantly enhances cell migration and promotes wound healing in osteoblasts[40], tubular epithelial cells[41], corneal epithelial cells[42], and fibroblasts[43]. However, the opposite effect was also found in a human prostate cancer cell line[44]. Together, it seems that the anti-apoptotic results of ROCK inhibition are more consistent than its effects on cell proliferation and migration among different laboratories[13–15, 28].

To date, only one study has shown Dox-induced apoptosis in endogenous CSCs of rat[17]. Our data indicated that Dox alone significantly induced cell death in a dose-dependent manner, but the expression pattern of the key apoptotic factors was not what was expected. Within the tested ranges (0 μM to 1.0μM), Dox induced a “Gaussian-type” response in protein expression for pAkt/Akt and Bcl-2, but a “Sigmoid-type” response for C-Caspase-3. The “Gaussian-type” dose-dependent response by Dox was also observed in tumor cell lines, such as human myeloma cells[36]. Sano *et al*.[37] also found that 0.3 μg/ml Dox (≈0.6μM) significantly increased the gene expression of Bcl-2 and Bcl-xl in a feline T-cell leukemia cell line while Lee *et al*.[45] reported a time-dependent “Gaussian-type” response of p-Akt in mouse NIH3T3 cells as well as in a human HaCat keratinocyte cell line following Dox incubation. Taken together, these studies indicate that short-time or low concentration exposure of Dox induces the upregulation of p-Akt and Bcl-2 in certain types of cells (*e*.*g*., tumor cell lines), but the biological importance of this response remains unknown. Tu *et al*.[36] suggested that Dox-induced upregulation of selective factors (such as Bcl-2 instead of Bcl-xl) in tumor cells may facilitate the increase in its drug resistance to a second exposure of Dox. In stem cells, a low Dox-induced increase in p-Akt and Bcl-2 may represent the same mechanism and biological role as in the tumor cells. It may simply reflect the initiation of a cellular protective mechanism when cells are exposed to low concentrations of Dox. Such protective responses could be decompensated at high concentrations of Dox due to its toxic effects.

The Dox-induced downregulation of p-Akt and Bcl-2 following Y-27632 pretreatment may involve the feedback activation of ROCK by cleaved Caspase-3[20]. Therefore, Dox-induced cellular responses following pretreatment of Y-27632 could be entirely different depending on the cell type tested and the underlying mechanisms, which may involve multiple signal transduction pathways. In the present study, four potential mechanisms were proposed (see Fig 8): (1) ROCK inhibition induces the downregulation of cleaved Caspase-3[24]; (2) ROCK inhibition induces the activation of ROCK to the p-MLC pathway and/or the inactivation of LIMK to the ADF/Coflin pathway[14, 46]; (3) ROCK inhibition induces the inactivation of Akt to the Bcl-2/Bcl-xl to Bax/Bak/Bim pathway; (4) ROCK inhibition induces the inactivation of p53 to the Bax/Bak/Bim pathway[46]. The present study supports the possible involvements of the first and second mechanisms in Y-27632-induced anti-apoptosis, but does not exclude other possibilities. Our findings and working hypothesis are in alignment with other published studies using distinct approaches, such as a different ROCK inhibitor (Fasudil) [33] or a ROCK1 gene knockout strategy[24, 47].

In conclusion, the present study demonstrated that pretreatment of Y-27632 significantly reduces Caspase-3 activity and protects CSCs from Dox-induced apoptosis. The underlying mechanisms are still not completely understood, and probably involve direct and/or indirect interactions between ROCK and Caspase-3, ROCK and LIMK/ADF/Cofilin, or ROCK and p-MCL (Fig 8). The overall consequence or balance among these interactions may result in anti-apoptotic effects on human CSCs. In spite of the fact that many questions remain unanswered, Y-27632 deserves further evaluation in stem cell-based therapy in animal models and the final results may be transferable to human clinical trials.
